# A bibliometric analysis of long non-coding RNA and chemotherapeutic resistance research

**DOI:** 10.18632/oncotarget.26938

**Published:** 2019-05-14

**Authors:** Xiaoman Chen, Yulu Shi, Kaiwen Zhou, Sijie Yu, Wei Cai, Muying Ying

**Affiliations:** ^1^Department of Molecular Biology and Biochemistry, Basic Medical College of Nanchang University, Nanchang, PR China; ^2^Queen Mary School of Nanchang University, Nanchang, PR China; ^3^Department of Medical Genetics and Cell Biology, Basic Medical College of Nanchang University, Nanchang, PR China

**Keywords:** chemotherapeutic resistance, lncRNA, citespace, bibliometric analysis

## Abstract

The global outputs of annual publication in long non-coding RNAs (lncRNAs) and chemotherapeutic resistance research exponentially increased from 2 in 2008 to 176 in 2017. Using Java application CiteSpace V and VOSviewer, this study assessed the publication model of lncRNAs and chemoresistance by bibliometric analysis. Totally, 2883 authors contributed 528 publications of lncRNAs and chemoresistance in 215 academic journals in the recent decade (2008-2018). Oncotarget in the 215 academic journals published the highest number of publications (60). China had the highest number of publication outputs (358). The leading institute was Nanjing Medical University. Wang Y was the most influential author (13 counts). Gupta RA had the most cited documents (87 counts). “Gene expression” and “poor prognosis” were identified as the hotspots. “Cancer stem cell”, “HOTAIR” and “UCA1” were the frontiers of the fields in recent years. The increase of publications on lncRNAs and chemotherapeutic resistance will continue in the next years. HOTAIR and UCA1 with multiple roles in drug resistance may offer big opportunities for targeted chemoresistance in cancer therapy. These results may help us discover and explain the possible underlying laws of the subject.

## INTRODUCTION

Chemotherapeutic agents, such as cisplatin, sunitinib, gemcitabine, paclitaxel and PARP inhibitors, have been widely applied in cancer treatment. While entirely eradicating the tumor and curing patients by chemotherapy alone are rare, in particular for advanced disease. The main obstacles are drug resistance that may be resulted from intrinsic and/or acquired factors. The factors of intrinsic resistance include drug breakdown, the aberrant expression and/or function of drug target, altered drug transport across membrane transporter, the ineffective interaction between drug and its molecular target [1]. Inactivation of tumor suppressor gene TP53 was also indicated to cause resistance to chemotherapeutic drugs [2]. Acquired drug resistance is usually associated with genetic or environmental factors that facilitate the development of chemoresistance cancer cell clones and/or induce gene mutations involved in relevant metabolic pathways. As the key regulatory factor of tumor stem cells, lncRNA Malat-1 can increase the ratio of pancreatic cancer stem cell, maintaining the ability of self-renewal, thus reduce the sensitivity to anticancer drugs [3]. LncRNA HOTAIR reprograms chromatin state to promote cancer metastasis [4]. The disorders or mutations of lncRNAs were indicated to be associated with cancer and chemoresistance, such as lncRNA CCAL in colorectal cancer [5], HOTTIP in osteosarcoma [6] and SNHG12 in non-small-cell lung cancer [7]. Publications focused on the research of lncRNAs and drug resistance rapidly increased in the past decade. Bibliometrics is a statistical method to assess various aspects (such as annual growth of publications, the contribution of countries, core journals and most active researchers) of scientific literature, which can help us discover and explain the possible underlying laws of the subject in lncRNAs and chemotherapeutic resistance research. There are still no reports about bibliometrics analysis of lncRNAs and chemotherapeutic resistance available in public database. Therefore, using Java application CiteSpace V [8], VOSviewer [9] and Microsoft Excel 2016 software, we carried out this study to qualitatively and quantitatively evaluate the published publications of lncRNAs and drug resistance for a better understanding on the various aspects of these publications.

## RESULTS

### Publication outputs and future development trend

**Figure 1:**
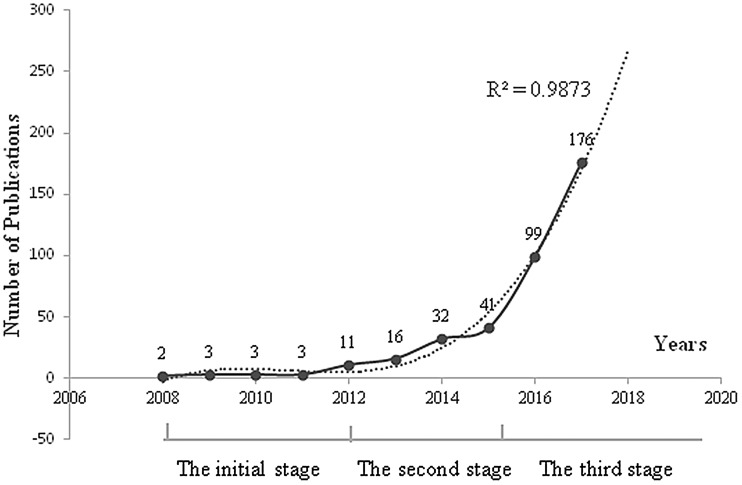
Publication outputs and growth prediction. The number of publications from 2008 to 2017 represented by the solid line; the dashed line represents the forecast curve, R^2^ = 0.9873.

Excluding 14 non-official articles or reviews (6: Book chapter; 4: Meeting abstracts; 2: Editorial material; 2: Proceedings paper), 528 publications (398: research articles; 130: review) indexed in the WoSCC of Thomson Reuters from 2008 to 2018 were extracted for further bibliometric analysis. 526 of the 528 articles (99.621%) were published in English, one in Chinese and one in German. The annual publications in WoSCC exponentially increased from 2 in 2008 to 176 in 2017 (Figure 1). With highly reliable correlation coefficients (R^2^ = 0.9873), the model fitting curve revealed a significant correlation between the annual publications and publication year. Based on the equation of model fitting, it is estimated that the number of publications on lncRNAs and chemotherapeutic resistance would be about 268 in 2018. And 243 publications indexed in the WoSCC were retrievable on August 19, 2018.

### Journal analysis

**Figure 2:**
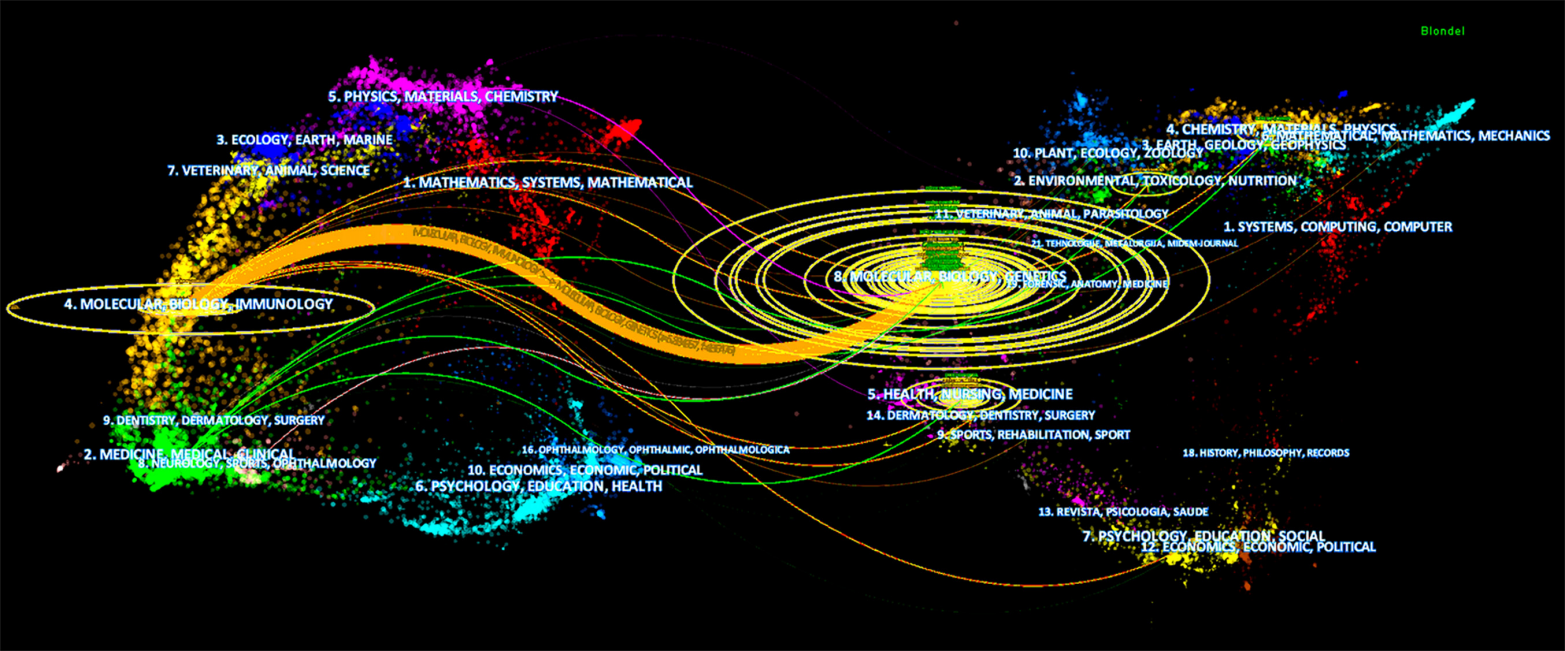
Dual-map overlay of journals on lncRNA and chemoresistance. Citation paths at a disciplinary level were demonstrated in t a dual-map overlay. The left of the map represented the cite journals and the right of the map represented the cited journals. Citation trajectories are colored based on the citing regions. The width of the paths is proportional to the z-score-scaled citation frequency.

The 528 publications of lncRNAs and chemoresistance were published in 215 academic journals. And the top 15 active journals published 206 of the 528 articles, accounting for 39.016% (Table 1). With 60 counts, Oncotarget published the highest publications of lncRNAs and chemoresistance, accounting for 11.364%, followed by Oncology Reports and Tumor Biology (Table 1). Citation paths at a disciplinary level were demonstrated in the visual representation called a dual-map overlay, which effectively mapped the field and direction of the topic (Figure 2). The labels on the map represented the subject fields covered by the journals. The left of the map represented where the collected reports published and the right regions rendered where it cited from (Figure 2). Most of these publications were published in molecular journals, biology journals, and immunology journals, and cited from molecular, biology, and genetics. Citing publications and cited publications are called research frontier and knowledge base, respectively. The base map consisted of the journal/conference-level citation relationships among over 1000 venues. Major clusters were labeled by terms chosen from the titles of venues in corresponding clusters. Citation trajectories were colored according to the citing regions. The width of the paths is proportional to the z-score-scaled citation frequency (Figure 2).

**Table 1:** The top active 15 journals that published these publications

Ranking	Journal	Country	Count	Percentage (%)	IF2017
1	Oncotarget	United States	60	11.364	5.168
2	Oncology Reports	United States	15	2.841	2.976
3	Tumor Biology	Switzerland	15	2.841	3.27
4	Cellular Physiology and Biochemistry	Switzerland	12	2.273	5.500
5	International Journal of Molecular Sciences	United States	12	2.273	3.687
6	Biomedicine and Pharmacotherapy	France	11	2.083	3.457
7	PLOS ONE	United States	11	2.083	2.766
8	Cell Death and Disease	England	10	1.894	5.638
9	Molecular Cancer	England	10	1.894	7.776
10	Oncology Letters	Greece	9	1.705	1.664
11	OncoTargets and Therapy	England	9	1.705	2.656
12	Biochemical and Biophysical Research Communications	United States	8	1.515	2.559
13	Cancer Chemotherapy and Pharmacology	United States	8	1.515	2.808
14	Cancer Letters	Netherlands	8	1.515	6.491
15	International Journal of Clinical and Experimental Medicine	United States	8	1.515	0.833

### Country and institution analysis

**Figure 3:**
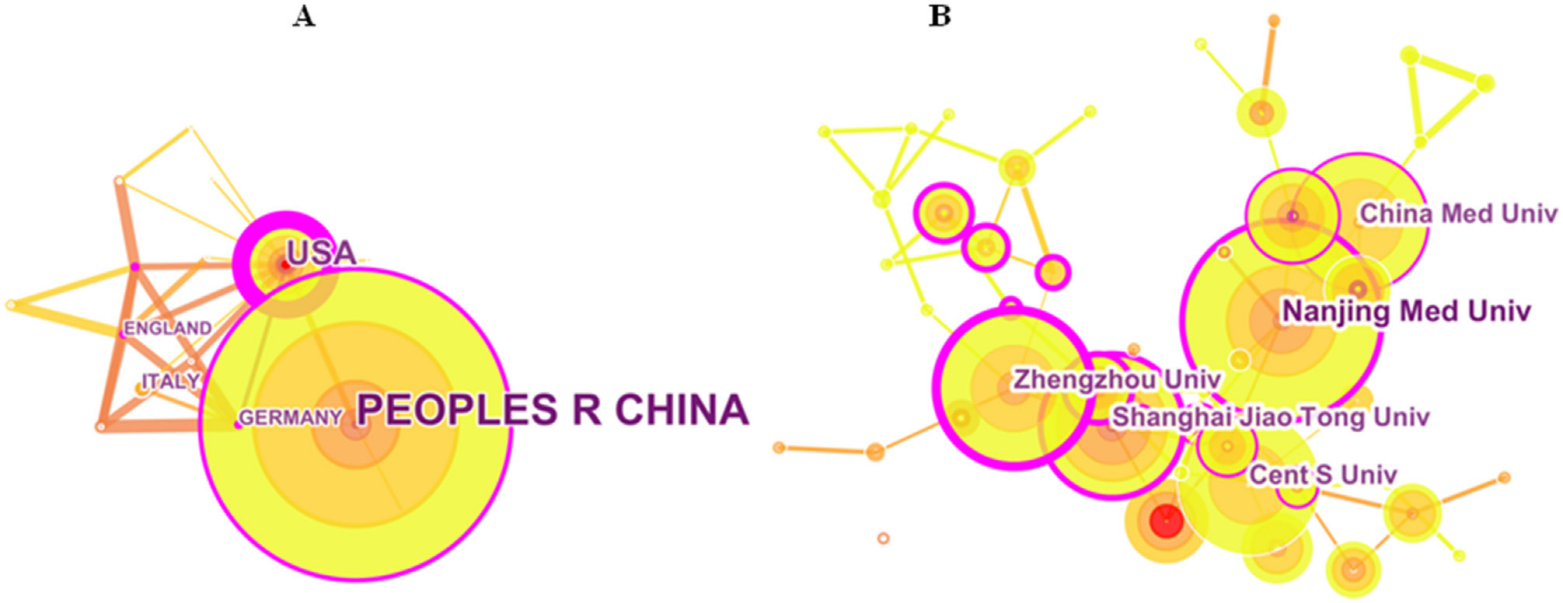
The distribution of countries/territories and institutions. (**A**) Map of countries/territories that published these publications. (**B**) Map of institutions that published these publications.

The distribution map of the country/institution can provide valuable information for researchers to better understand the community where they’re working. The distribution map indicated that the 528 publications came from 695 research institutes in 46 countries/territories (Figure 3A). The top 10 countries/territories that contributed these publications included three Asian countries, three European countries, two North America, one Middle East and one Oceania (Figure 3A and Table 2). China (358) and the United States (98) were the top two countries, accounting for 86.364% of the total publications (Figure 3A). As a developing country in this group, China had made remarkable progress in this field in the past decade. While the United States ranked the first in the citation frequency. Therefore, for the quality of research, the United States was the leading nation in this field. The cooperation between the United States and other countries was much closer than that of China (Figure 3A).

**Table 2:** The top ten productive countries and institutions of the total publications

Ranking	Country	Count	Institution	Count
1	China	358	Nanjing Medical University	29
2	USA	98	Zhengzhou University	22
3	Italy	21	Central South University	21
4	Germany	17	Shanghai Jiao Tong University	21
5	England	14	China Medical University	20
6	Australia	9	Nanjing University	14
7	Japan	9	Tongji University	13
8	Taiwan	9	Dalian Medical University	13
9	Canada	6	Harbin Medical University	11
10	Iran	6	University Texas MD Aanderson Cancer Center	10

The top 10 institutions published 174 of the 528 publications, accounting for 32.955% (Table 2). Nanjing Medical University (29) ranked first place, followed by Zhengzhou University (22) and Central South University (21) (Figure 3B and Table 2). Among the top 10 active institutions, one was from the United States (The University of Texas M.D. Anderson Cancer Center) and 9 were from China. Chinese institutions were in relatively close cooperation in the collaboration network, which may partially explain the observation that China contributed to most of the publications.

### Author analysis

The 528 articles were written by 2883 authors working in the subject. The network map embodied the cooperation among authors (Supplementary Figure 1A). Among the top 10 authors with the largest number publications, WANG Y (13 publications) ranked the first, followed by LI J (11 publications) and Liu Y (11 publications) (Supplementary Figure 2 and Table 3). The author citation information was presented as a co-cited author map (Supplementary Figure 1B). Among the top 10 authors with the largest citation, Gupta RA was the top-ranked author (87), followed by Wang Y (79) and Mercer TR (58) (Table 3).

**Table 3:** The top 10 authors and co-cited authors working in the field

Ranking	Author	Count	Co-cited Author	Count
1	Wang Y	13	Gupta RA	87
2	Li J	11	Wang Y	79
3	Liu Y	11	Mercer TR	58
4	Li X	9	Gutschner T	55
5	Wang J	8	Fan Y	51
6	Wang L	7	Wang F	48
7	Li L	7	Tsang WP	47
8	Li Y	7	Wang KC	46
9	Zhang L	7	Li J	43
10	Shang C	6	Jemal A	38

### Reference co-citation analysis

**Figure 4:**
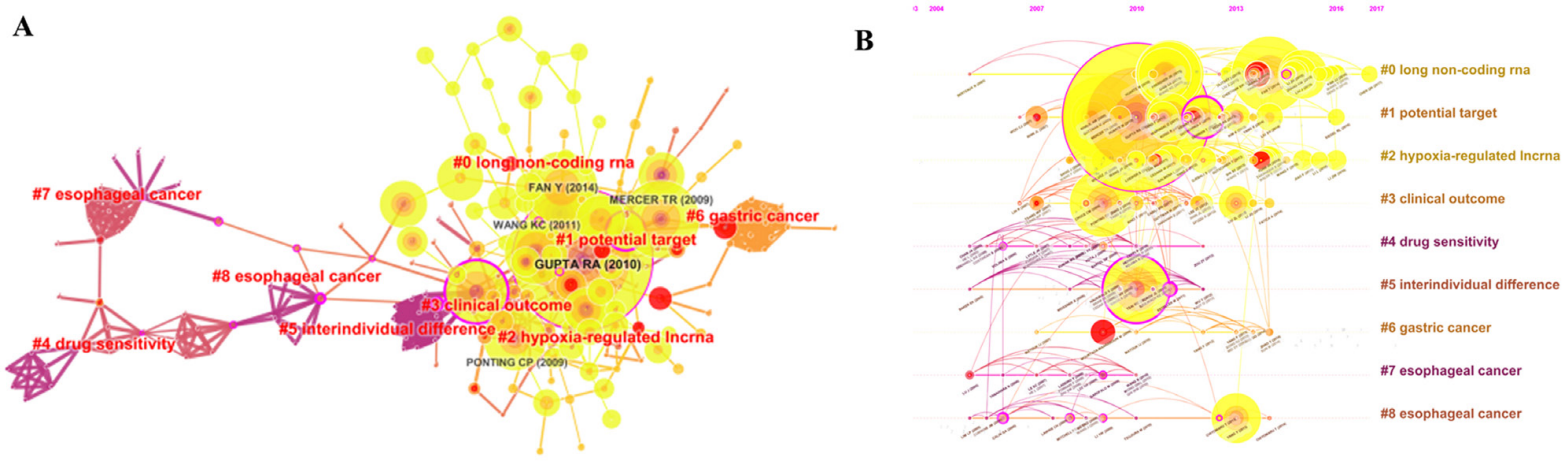
The analysis of references. (**A**) Co-citation knowledge map of references on lncRNA and chemoresistance. (**B**) Timeline view of references on lncRNA and chemoresistance with log-likelihood ratio (LLR) cluster labels. The color of the node represents the newness of the related reference (the red color for new studies).

The knowledge map of citation reference of the 528 publications with 258 nodes and 539 connection lines was built (Figure 4A). The knowledge map was composed of centrality and citations, which can be used to analyze the frontier areas of this field by exploring the key clusters of publications (Figure 4A). Each cluster is a distinctive specialty or a thematic concentration. The modularity Q score was calculated to evaluate the modularity of the clustering network. The modularity Q score ranges from 0 to1, where a high value indicates that the object is well matched to the modularity. The modularity value was 0.7882, indicating that the network was reasonably divided into loosely coupled clusters. Preliminary analysis showed 51 clusters with a Mean Silhouette of 0.3891. Filtered out small clusters with a low silhouette (less than 10), the top 8 largest clusters were finally retained. Among the 8 clusters, the largest cluster #0 was “long non-coding RNA”, followed by cluster #1, labeled as “potential target”, and the third largest cluster #2 labeled as “hypoxia-regulated LncRNA “. Figure 4B was a timeline view of the top 8 largest clusters that showed the citations of each document in each cluster, and the corresponding changes of the key clusters.

### Keyword co-occurrence cluster analysis

**Figure 5:**
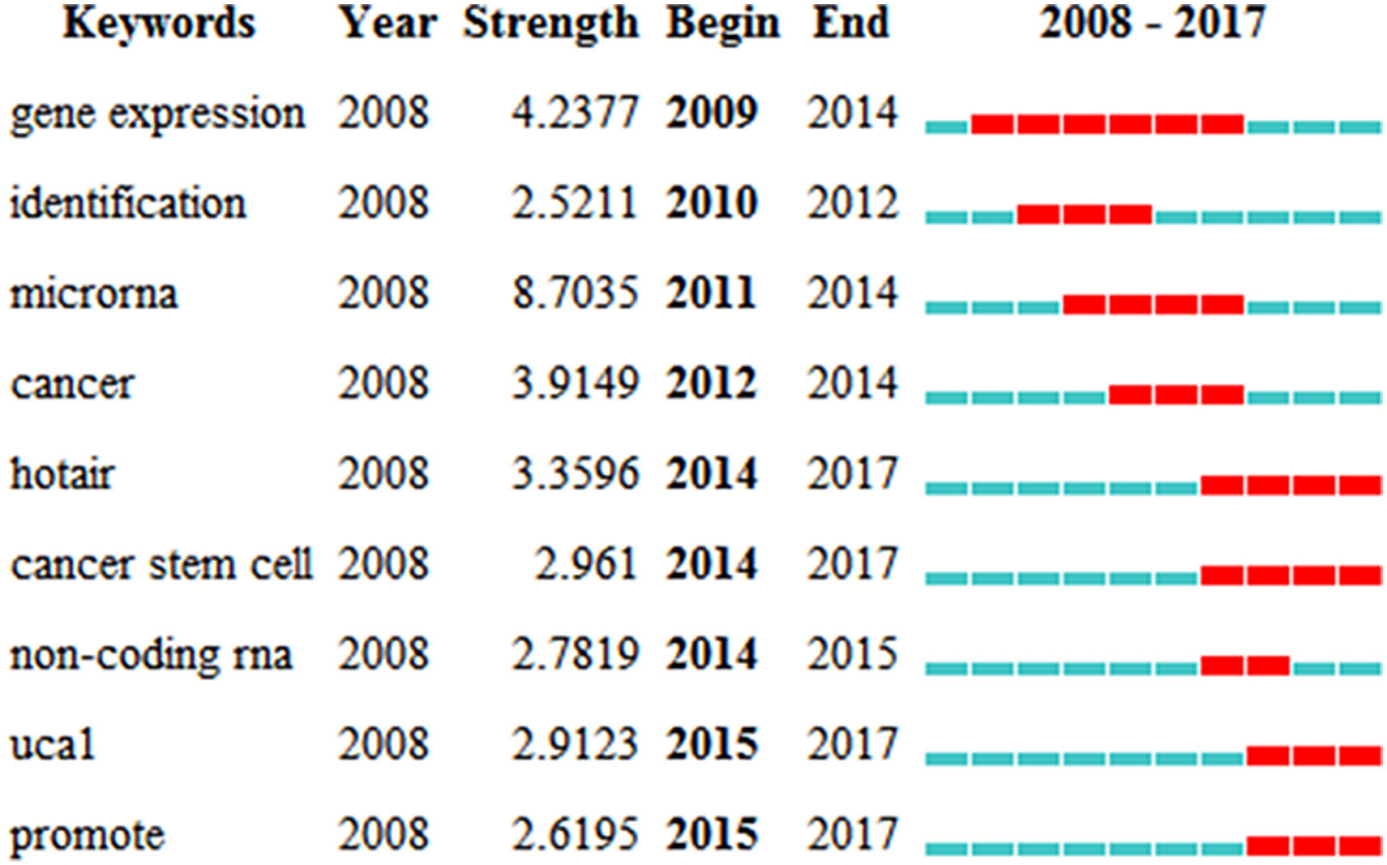
The top 9 keywords with the strongest citation bursts.

Keywords with high degree centrality and frequency represent the research hot spots over a period of time. The burst keywords represent new research frontiers. Centrality is an indicator of the relative importance of nodes in a network with respect to the total system activity. The higher frequency of co-occurrence of keywords and the stronger centrality of the points indicate the more important of the node in the field [10]. Among the 128 keywords (Indication and Class Description) extracted from the 528 publications, the top 3 high-frequency keywords were “long non-coding RNA”, “expression” and “drug resistance” (Supplementary Figure 2). The histogram showed 14 of the top 17 high-frequency keywords, excluding drug resistance, chemoresistance and chemotherapy, which were roughly divided into three parts: “gene expression”, “poor prognosis” and “cancer types” (Supplementary Figure 3). Among the 9 burst keywords, the strongest ones after 2014 included “HOTAIR” (2014–2017), “cancer stem cell” (2014–2017), “non-coding RNA” (2014–2015), “UCAL” (2015–2017) and “promote” (2015–2017) (Figure 5).

## DISCUSSION

### Co-existing analysis

The analysis of publication outputs indicated that the development of lncRNAs and chemoresistance research could be roughly divided into three stages: the initial stage (2008–2011) developing at a slow pace (2 in 2008; 3 in 2009, 2010 and 2011, respectively); the second stage with steady growth (2012–2015) (11 in 2012; 16 in 2013; 32 in 2014; 41 in 2015); the third stage with exponential growth (after 2015) (Figure 1). The prediction curve showed that the growth accompanied with the increase of our knowledge in the subject will continue in the next few years.

The impact factor (IF) of a journal is an important factor to evaluate its influence of the published articles. Among the top 15 active journals that published these publications, 6 journals had an IF > 3.0, accounting for 30.583% (6/15). Four journals (4/15, 19.417%) had an IF > 5 (Table 1). Journals with extremely high impact factor, such as Nature, Science and Cell, did not publish the topic articles. Maybe, these publications that studied the roles of lncRNA in chemotherapeutic resistance were not enough to be published in extreme high-impact journals, and thus we still need to strive for excellence in this field.

### References and citation analysis

Everyone in the top 10 prolific authors had at least six publications. While the comparison of the top 10 prolific authors with the top 10 co-cited authors showed that only Wang Y and Li J in the 10 prolific authors also appeared in the list of the top 10 co-cited authors (Supplementary Figure 1 and Table 3). The observations may suggest that authors should pay more attention to the quality of their papers when trying to increase their publication number.

The analysis of references showed that Gupta RA (2010) had the highest number of references (87), followed by Mercer TR (2009) with 50 references and Fan Y (2014) with 45 references (Figure 4A). The timeline graph revealed the high-impact articles published in 2009–2016. The largest cluster #0 labeled as “long non-coding RNA” indicated the high co-citation articles for each year (Figure 4B). Analysis of high co-citation articles in cluster #0 revealed the diverse biological functions of lncRNAs. For example, as a part of the complex, lncRNA H19 associated with EZH2 enhancer and inhibited E-cad expression, which resulted in the activation of the Wnt/β-catenin pathway enhancing bladder cancer metastasis [11]. The increased level of lncRNA UCA1 activated Wnt signaling and resulted in the cisplatin resistance of bladder cancer cells in a Wnt6-dependent manner [12].

### Research hot spots

Keywords are the author’s generalization and refinement of the article core. Keywords with a high degree centrality were “gene expression”, “poor prognosis” and “cancer types”, indicating that research topics represented by these keywords are at the core of lncRNA and chemotherapeutic resistance research in the past decade (Supplementary Figure 3). To date, about 20 lncRNAs were demonstrated to mediate chemotherapeutic resistance by various and often undefined mechanism in different cancer [13]. Through competitively binding mR-33a-5P, miR-33b-5P, miR-1-3P, miR-206 and miR-613, ANCR increased AXL expression and activated PI3K/Akt/NF-κB signaling pathway that caused cisplatin resistance in glioma cells [14]. Breast cancer patients with poor response to chemotherapy had fourfold higher CRALA level than responding patients [15]. Chronic oxymatrine treatment induced chemotherapy resistance and epithelial-mesenchymal transition through targeting MALAT1 in colorectal cancer cells [16]. The increased expression of HOTAIR and its DNA methylation appeared to induce carboplatin resistance [17], indicating that HOTAIR may contribute to resistance by influencing methylation.

### Research frontiers

The burst words represent keywords that is often cited over a period of time, thereby indicating the frontier areas. The first 3 burst words after 2014 were “cancer stem cell”, “HOTAIR” and “UCA1” (Figure 5). Cancer stem cell is cancer cells that possess the characteristics of normal stem cells and can generate tumors through the stem cell processes of self-renewal and differentiation into multiple cell types. LncRNAs known to be a regulatory factor of tumor stem cells included GAS5, NEAT1, SOX2OT, Malat-1, HOTAIR, PVT1, BCAR4 and RBM5-AS1. As the key regulatory factor of tumor stem cells, Malat-1 increased the ratio of pancreatic cancer stem cell, maintained the ability of self-renewal, and thus reduced the sensitivity to anticancer drugs [3].

The burst words of “HOTAIR” and “UCA1” reflected a wide study of their roles in chemoresistance from the scientific community. HOTAIR has been shown to mediate radiosensitivity, endocrine resistance and chemotherapeutic resistance in a variety of ways [18]. The increased HOTAIR expression induced cisplatin resistance through activatingthe Wnt/b-catenin pathway and promoting cell cycle progression in ovarian cancer [19]. HOTIAR mediated chemoresistance in DNA-damage responses through the NF-κB signaling pathway [20]. Both *in vitro* and *in vivo* experiments confirmed that UCA1 induced cisplatin/gemcitabine resistance through the activation of CREB by p-AKT and the subsequent up-regulation of miR-196a-5p [21]. The increased UCA1 activated the Wnt pathway and decreased cisplatin-induced cell death [12]. UCA1 in SKOV3 cells increased SRPK1 and Bcl-2, decreased Bax, Caspase-3 and Caspase-9 expression and contributed to cisplatin resistance [22]. UCA1 induced tamoxifen resistance in breast cancer cells partly through activation of the mTOR pathway [23]. Acting as a sponge for miR-18a, UCA1 is an important modulator of tamoxifen resistance by regulating cell cycle proteins [24]. The multiple roles of HOTAIR and UCA1in drug resistance may offer big opportunities for the targets of cancer therapy.

## MATERIALS AND METHODS

### Data collection

The publications were retrieved on Web of Science Core Collection (WoSCC) of Thomson Reuters on August 19, 2018. The search query consisted of seven terms as follows: (TS = (chemoresistance OR chemotherapy resistance^*^ OR chemotherapy drug resistance^*^ OR chemotherapy-refractory drug^*^ OR chemotherapeutic drug^*^ OR drug resistance^*^)) AND (TS = (lnc RNA^*^ OR long ncRNA^*^ OR long non translated RNA^*^ OR long non-coding RNA^*^ OR long non protein coding RNA^*^ OR long non-coding RNA^*^ OR long untranslated RNA^*^ OR long intergenic non protein coding RNA^*^ OR large intergenic non coding RNA^*^ OR large intergenic noncoding RNA^*^ OR lincRNA^*^ OR linc RNA^*^)) The wildcard character “^*^” captures any relevant variations of a term such as bibliometrics and bibliometric analysis. A bibliographic record was considered as relevant if any of the terms appear in its title, abstract, or keywords. The query returned 542 bibliographic records written in English between 2008 and 2018 on August 19, 2018. Excluding 14 non-official articles or reviews (6: Book chapter; 4: Meeting abstracts; 2: Editorial material; 2: Proceedings paper), 528 publications (398: research articles; 130: review) were retained for further analyzed. The pipeline of the research procedure was displayed in Supplementary Figure 4.

### Investigating the intellectual structure in bibliometrics

The publication records, including author, title, abstract, organization, country, journal, keyword, and reference, were extracted and input into CiteSpace [8] in txt format to build the knowledge network, map historical footprint and investigate citation paths at a disciplinary level. Given the records, the toolkits detect and render thematic patterns and emerging trends in science as networked in a variety of bibliographic units. Using a top-down approach, we analyzed data going from macro-level to micro-level, which allowed us to gradually move on to lower-level units of analysis such as journal-level citation paths, subject categories, keywords, titles and abstracts to cited references. In doing so, we effectively captured emerging trends, recent developments and current problems in the research of ncRNA and chemoresistance. At the same time, Microsoft Excel 2016 was used to predict the future trend of LncRNA and chemotherapeutic resistance publications, The model: *f*(*x*) = *ax*^3^ + *bx*^2^ + *cx* + *d*, *x* represents the publication year, *f*(*x*) represents the cumulative number of publications.

The strategies in the analysis of these publications included: (1) Clustering: Clustering is unsupervised learning that can uncover latent groups of entities sharing homogeneous characteristics. We captured thematically similar clusters on a document co-citation network by using a network clustering technique called smart local moving [25] to (2) Burst detection: burst detection models the burstiness of features which rise sharply in frequency [26]. An entity has bursting activities when it intensively appears during a specific span of time. We can overcome the limitation coming from considering cumulative, snapshot metrics as impact measures; (3) Cluster labeling: CiteSpace labels clusters with extracted terms from titles and abstracts of citing articles. There are three algorithms to serve cluster labeling: latent semantic analysis (LSA), log-likelihood ratio (LLR) and mutual information (MI). LSA captures unknown semantic relationships over all the documents while LLR and MI reflect a unique aspect of a cluster [27].

## SUPPLEMENTARY MATERIALS


