# Supplementary material for: A bibliometric analysis of long non-coding RNA and chemotherapeutic resistance research
Source: Oncotarget. 2019 May 14;10(35):3267–75. doi: 10.18632/oncotarget.26938 (PMC6524938; doi:10.18632/oncotarget.26938)
Supplement: Supplementary file 1 [file oncotarget-10-3267-s001.pdf]

A bibliometric analysis of long non-coding RNA and chemotherapeutic resistance research

SUPPLEMENTARY MATERIALS

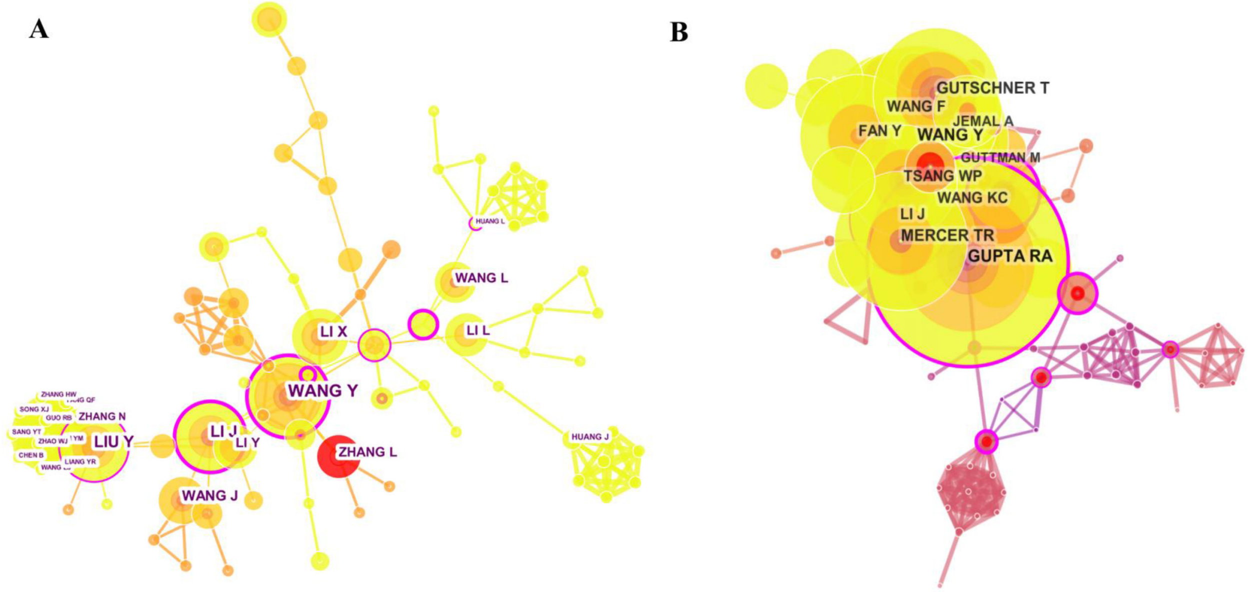

Supplementary Figure 1: The analysis of authors and co-cited authors of these publications. (**A**) Author analysis: the network map embodied the cooperation among authors. (**B**) Network map of author co-cited analysis.

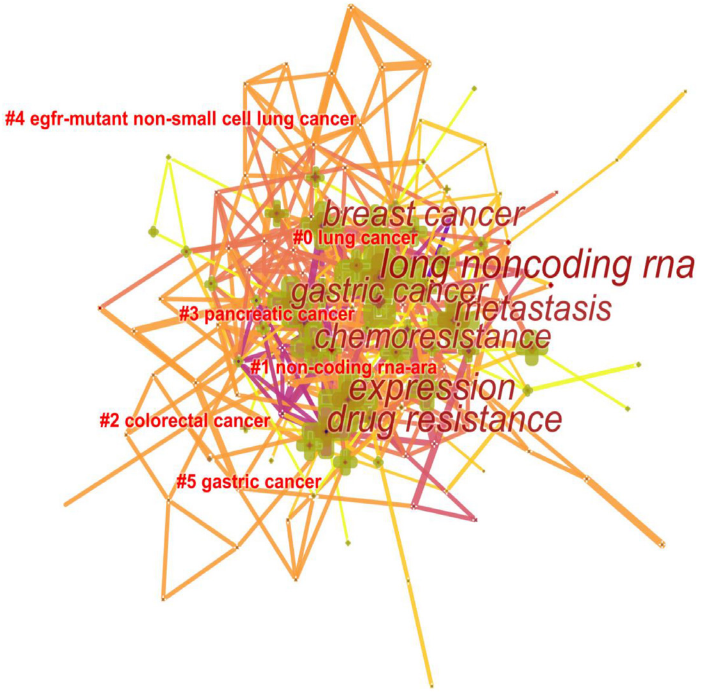

Supplementary Figure 2: The knowledge map of keyword co-occurrence. The top 3 high frequency keywords are “long non-coding RNA”, “expression”, and “drug resistance”. There were initially 6 clusters, including #0 lung cancer, #1 non-coding RNA-ARA, #2 colorectal cancer, #3 pancreatic cancer, #4 egfr-mutant non-small cell lung cancer, #5 gastric cancer.

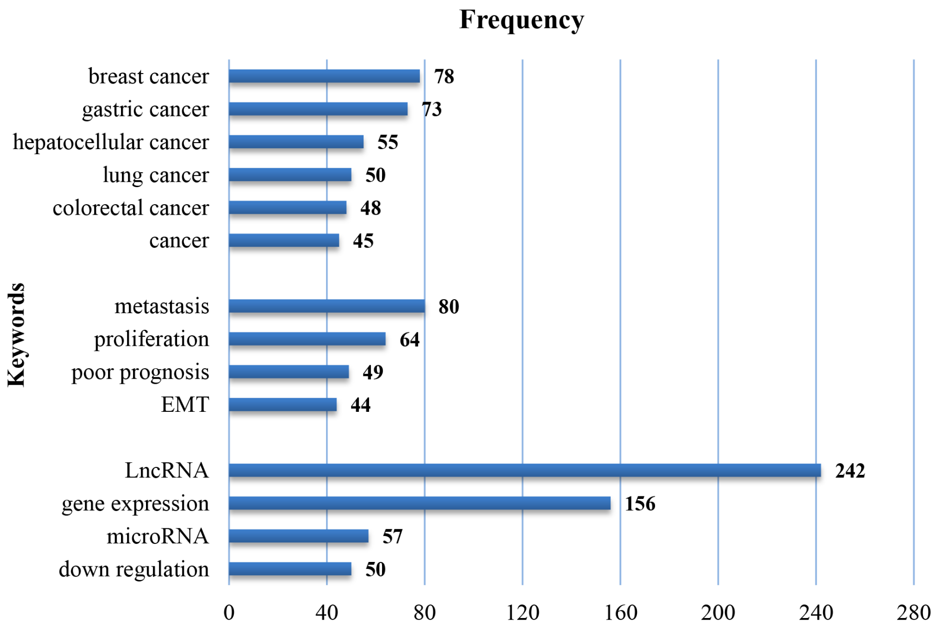

Supplementary Figure 3: The analysis of research hot spots. In addition to drug resistance, chemoresistance and chemotherapy, 14 high-frequency keywords can be divided into three categories: "poor prognosis", "cancer kinds" and "expression". X axis: the citation frequency of high-frequency keywords; Y axis: high-frequency keywords; EMT: Epithelial–mesenchymal transition.

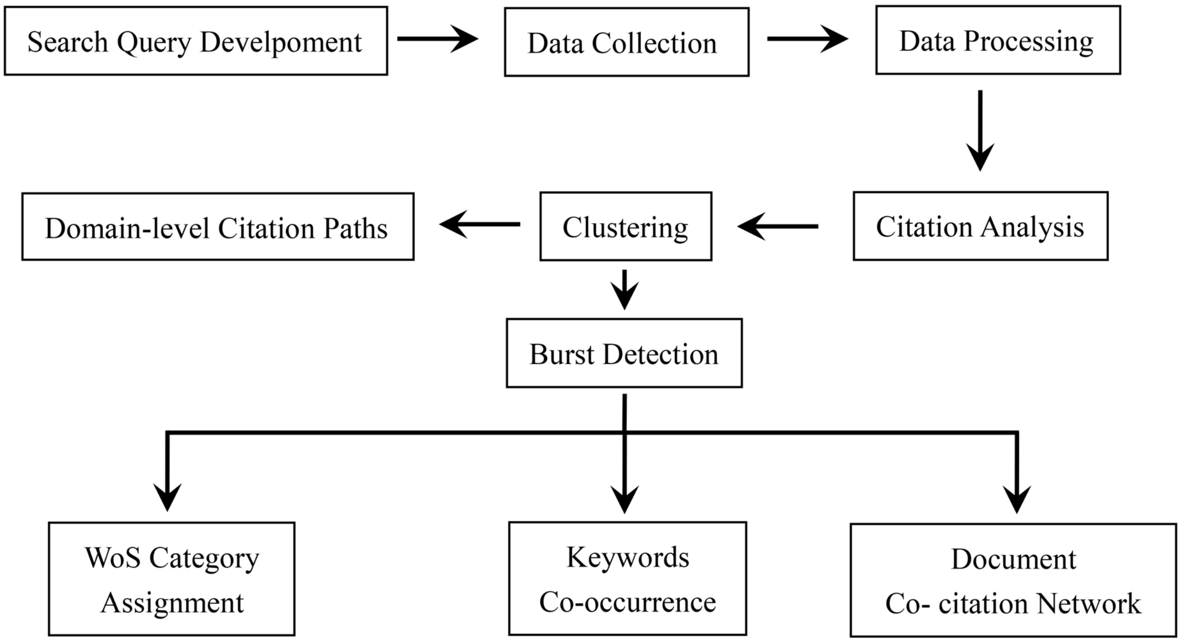

Supplementary Figure 4: The pipelines of the research procedure.
